# Respiratory symptoms after TB treatment completion: A qualitative study of patient and provider experiences in urban Blantyre, Malawi

**DOI:** 10.1371/journal.pgph.0003436

**Published:** 2024-09-27

**Authors:** Jamilah Meghji, Wezi Msukwa-Panje, Elizabeth Mkutumula, Wala Kamchedzera, Ndaziona P. K. Banda, Peter MacPherson, Nora Engel

**Affiliations:** 1 National Heart and Lung Institute, Imperial College London, London, United Kingdom; 2 Malawi Liverpool Wellcome Trust, Blantyre, Malawi; 3 Department of Medicine, Kamuzu University of Health Sciences and Queen Elizabeth Central Hospital, Blantyre, Malawi; 4 School of Health & Wellbeing, University of Glasgow, Glasgow, United Kingdom; 5 Clinical Research Department, London School of Hygiene & Tropical Medicine, London, United Kingdom; 6 Department of Health, Ethics & Society, Maastricht University, Maastricht, Netherlands; Weill Cornell Medicine, UNITED STATES OF AMERICA

## Abstract

Pulmonary tuberculosis (PTB) survivors experience a high burden of residual and recurrent respiratory symptoms after TB treatment completion. However, guidelines for the investigation and care of symptomatic TB-survivors are limited. We used qualitative methods to explore patient and provider understandings, experience and practice around respiratory symptoms in the post-TB period. We conducted in-depth interviews with PTB-survivors who had experienced respiratory symptoms (cough, chest pain, breathlessness) after successful TB treatment completion in Blantyre, Malawi (n = 23). We completed focus group discussions with TB-Officers (n = 12), and in-depth interviews with health care workers (n = 18) from primary and tertiary health facilities. Interviews were conducted in Chichewa, and thematic analysis was used to identify common themes. Our data highlight that TB survivors have negative experiences of respiratory symptoms after TB treatment completion, with anxiety about the cause of symptoms, uncertainty about if and how to return to care, and fear of recurrent TB disease. Our findings suggest four critical practices which shape this experience including: limited counselling at TB treatment completion; the lack of clear health seeking pathways to return to care; the use of TB-focused investigations for those returning to care; and heterogeneous approaches to TB retreatment decisions. This study highlights that the post-TB period is a critical part of the patient’s experience of TB disease. Current practices create a negative patient experience, and carry clinical and public health risks including delayed diagnosis of TB relapse, missed diagnosis of cardio-respiratory disease, and misuse of antimicrobials and TB retreatment. Formative guidelines are needed to improve the care of symptomatic TB-survivors.

## Background

An estimated 10.6 million people experienced tuberculosis (TB) disease in 2022, and although survival is improving, for many individuals health does not return to normal at TB treatment completion. TB-survivors, thought to number at least 155-million in 2020 [1], face a high burden of residual morbidity and have increased mortality rates compared to people who have never been treated for TB disease [2, 3]. It is estimated that half of the disability adjusted life years (DALYs) lost due in relation to TB disease occur in the post-TB period [4, 5].

Post-TB lung disease (PTLD) is a major contributor to post-TB morbidity. Residual lung function abnormalities, imaging changes, and respiratory symptoms are seen in between 40–60% of pulmonary TB (PTB) survivors [6], and PTLD is associated with reduced quality of life, ongoing health seeking, and reduced income and employment amongst PTB-survivors [7–9]. However, there remain few guidelines or framework for the clinical management of TB-survivors with PTLD.

TB-survivors are also at risk of recurrent TB disease–in high TB incidence settings TB-survivors have been shown to be at increased risk of incident TB disease compared to TB naïve adults [10, 11], and a recent TB prevalence survey from urban Malawi showed that although TB notifications and prevalence are falling, a history of TB disease remains a strong predictor of prevalent disease [12]. TB-survivors may also have other forms of chronic lung disease related to exposures such as tobacco smoking, occupational exposures, or drug use [13–15]. The challenge of differentiating between PTLD, recurrent PTB disease, and broader cardio-respiratory diseases amongst symptomatic TB-survivors returning to care is well recognised by health care providers in low-resource settings, and high rates of empirical TB retreatment have been observed for those with intractable respiratory symptoms [16].

However, little is understood about current approaches to the management of symptomatic TB-survivors. Qualitative research around TB care has largely been focused on health seeking and care during TB illness and disease, with little attention on the post-TB period [17], and as a result little is known about how TB-survivors and health care workers understand or approach residual or recurrent respiratory symptoms. Previous studies have highlighted the fear of recurrent TB disease and treatment experienced by many TB survivors, and the stigma and uncertainty arising from a diagnosis of recurrent disease [18]. However, the majority of national TB guidelines, including those used in Malawi, do not include formal guidance around morbidity screening at treatment completion, or include linkage to / the provision of ongoing care [19].

In this qualitative study, we investigated the experiences and practices of TB-survivors and health care workers around residual or recurrent respiratory symptoms in urban Malawi. Our aim was to understand how these symptoms are understood, interrogate approaches to health seeking, clinical investigation and management, and to describe patient and provider experiences of care.

## Methods

Focus group discussions (FGDs) and in-depth interviews (IDIs) were completed with health care workers (HCW) and TB-survivors in urban Blantyre, Malawi between 11th January and 13th December 2022.

### Recruitment

TB-survivors aged ≥18-years who had successfully completed treatment for PTB disease, but reported chronic or recurrent respiratory symptoms (cough, chest pain, breathlessness, sputum production), were recruited for IDIs. We recruited both individuals who were and were not engaged with health seeking, and recruitment was stratified by HIV status, which is recorded in the majority of TB patients in Malawi.

Health facility sampling was used to identify those who were actively engaged in health seeking. TB-Officers (TBOs)–a cadre of environmental health officers who deliver TB services in Malawi–from the five largest health facilities in Blantyre (Queen Elizabeth Central Hospital (QECH), and Ndirande, Limbe, Bangwe and Zingwangwa Health Centres) were asked to identify and refer sequential patients attending health centres for recurrent respiratory symptoms, whether or not the patient went on to re-initiate TB treatment. Community sampling was used to identify those who were not actively engaged in health seeking, using two approaches designed to capture TB survivors at a range of times from treatment completion. Firstly, a random sample of PTB-survivors who had completed treatment in Bangwe district within the past 2–5 years was taken from the district TB register. These individuals were contacted by phone and approached to participate if they had ever experienced respiratory symptoms after TB treatment completion. Secondly, PTB-survivors reporting residual respiratory symptoms at TB treatment completion were identified from a morbidity surveillance study which was being run concurrently in urban Blantyre, by our team. These individuals were sequentially contacted by phone 6–12 months after treatment completion and approached to participate.

A convenience sample of TBOs from 18 health centres across Blantyre was recruited for participation in two FGDs. Participants were identified at the monthly TB surveillance meetings held for TBOs in Blantyre, and FGDs were held alongside these meetings. Previous experience of qualitative work suggests that TBOs are comfortable sharing their experiences around patient care in group discussions [20].

Facility-based health care workers (HCWs) were purposively recruited for IDIs. The initial sample included TBOs who were engaged in the FGDs, and HCWs who were known by the study team to be involved in TB and respiratory care in Blantyre. A snowballing approach was then used to identify others with interest and experience in managing symptomatic TB-survivors, with purposive sampling to include providers with diverse levels of training and experience.

### Data collection

Interview topic guides were developed using existing literature on TB and chronic respiratory disease, and were rooted in previous stakeholder engagement work with TB patient groups, health care providers and policy makers from East Africa [21]. Topics explored with TB-survivors included: experiences of receiving and completing TB treatment; perceptions of health and wellbeing after treatment; the meanings ascribed and actions resulting from residual or recurrent respiratory symptoms; drivers and experiences of health care seeking for these symptoms; perceptions of risk around recurrent TB; and experiences of re-treatment. Topics explored with HCWs included: current practice at TB treatment completion; beliefs about the meaning and drivers of residual or recurrent symptoms; approaches to the investigation of those returning to care, including the use of TB and respiratory diagnostics; approaches to decision making around care, including antibiotic use and TB retreatment. Both TB-survivors and HCWs were asked about perceived challenges in care for symptomatic TB-survivors, and for their suggestions of how to address these.

Written informed consent was obtained from all participants, with support from a guardian or impartial witness for those unable to read. FGDs and IDIs were conducted in Chichewa, the local language spoken in Blantyre, Malawi, by fluent research assistants (WMP and EM). Interviews and focus groups were held at sites where confidentiality could be maintained, and which were comfortable to or chosen by the participants. This included locations outside, in participant’s homes, or at local health facilities. Discussions were audio-recorded and transcribed and translated into English. The research team kept notes of interview findings and contexts, with memos used to support reflexivity about the perspectives and position of the research team. Debriefings between the research team members took place regularly to share insights and observations, discuss emerging themes and relationships between them, and iteratively review the topic guide. Final sample size was determined by saturation, with initial plans for IDIs with up to 20 HCWs and 40 TB-survivors.

### Data coding and analysis

Data were imported into NVivo (QSR International) for coding. Broad codes were developed for HCW and TB-survivor data. This was done both deductively and inductively–initial codes were based on the research questions and interview topics, and were adapted over time in response to the data and as more refined sub-codes emerged. Coding was led by the research assistant who had led data collection (WMP) with parallel coding of early transcripts and ongoing review by JM and EM. Discussion with the broader research team was used to reconcile coding differences (JM, WMP, EM, NE). Thematic analysis was employed to identify patterns, themes, and key findings across the HCW and TB-survivor data together. This was an iterative process involving several members of the research team (JM, WMP, EM, NE), and required moving back and forth between the coded material, researcher observations and memos, and emerging analysis, and theoretical approaches to understand the data [22]. We borrowed from theories on the TB care cascade [23], and the invisible work of patients and health care workers in completing diagnostic journeys to support this analysis [24, 25]. We employed a technology-in-practice perspective which recognises that medical practice is a result of diverse elements including bodies, samples, equipment, the physical organisation of clinical spaces, and conversations and relationships between those receiving and providing care [26]. Data from HCWs and TB survivors were compared to support triangulation.

The characteristics and qualifications of the research team, and factors affecting research reliability, validity and transferability are described in the Supplementary materials (S1 and S2 Tables).

### Ethical approvals

All participants provided written informed consent. Ethical approval was obtained from the Liverpool School of Tropical Medicine (21–069) and Malawi College of Medicine Research Ethics (P.08/21/3377) Committees.

## Results

We conducted IDIs with 23 TB-survivors identified from health facilities (n = 13) and through community sampling (n = 10). We completed IDIs with 18 HCWs including TB-Officers (n = 6), health facility nurses (n = 2), clinical officers (n = 6), and medical doctors (n = 4). We conducted two FGDs with 12 TB-Officers in total. Interviews lasted up to an hour, and the focus group discussions lasted approximately an hour each.

### Patient experience of residual or recurrent symptoms

TB-survivors reported negative experiences of residual or recurrent symptoms after TB treatment completion, dominated by themes of uncertainty, fear, and anxiety.

Participants with residual symptoms at treatment completion expressed significant uncertainty, asking why they did not feel better, and whether the TB medication had worked (Table 1, Quote 1). Some sought reassurance from TB-Officers that they had been cured (Table 1, Quote 2), with others pursuing repeat CXR imaging to understand their health and obtain ‘peace of mind’ (Table 1, Quote 3). The concept of post-TB lung disease–described as ‘sores’ within the lung–was frequently used by TB-Officers to explain residual symptoms to patients, but was framed as a temporary problem which would heal over time rather than a chronic condition. For those that failed to improve there was a strong mismatch between the reassurance received and their own experience of ongoing poor health, and this led to further uncertainty and loss of faith in the care received (Table 1, Quote 4). Participants expressed uncertainty about whether, when and how to seek care for symptoms, reporting a lack of guidance from TB-Officers at treatment completion. (Table 1, Quote 5).

**Table 1:** Quotations.

Section	Number	Quote
Patient experience	1	“My concern is coming in because I am not getting better despite taking the medication. I am partly well and partly unwell because I am still coughing. I have no peace inside me because of this.” (TB-survivor, Health facility)
	2	“Some of those with pulmonary TB (…) have developed post TB complications, maybe shortness of breath is continuing or maybe they are still experiencing chest pains, they worry that despite the fact that they have taken the drugs for six months, they are still experiencing the signs and symptoms as reported (…) the only thing that is required is to reassure them and explain properly the situation that they are in, informing them that they cannot just get completely cured in an instant and they should acknowledge that they will experience some problems.” (TB-Officer, Focus group discussion)
	3	“They said, ‘You have passed. Things are well.’ but I had an interest to undergo an X-ray, I went there alone on that van that gets stationed outside the gate (…) it was better to see the pictures again, to see how my chest looks. This would free me.” (TB-survivor, Health facility)
	4	“I was told that it could be possible that the sores were not completely healed, but after the sores clear out you will feel better, and this would take some time to normalise. After getting this assurance from the doctors, I went back home. (…) I realised that I was becoming weaker and weaker (…) I could see my body getting deteriorated, but I was being told that the sores had not healed and I could ask myself: they are saying that the sores had not healed, and yet my body is becoming weaker, what is my hope on this as of now?” (TB-survivor, Health facility)
	5	“I wasn’t given any advice after finishing my treatment there. They had to ask me if I got the [TB sputum] results… but I was not given any advice.” (TB-survivor, Health facility)
	6	“I remember this other time, I was asked to go to the traditional healer, but I refused to take this advice. Of course I [knew that I] was going to be diagnosed with TB someday and not that I was bewitched by someone” (TB-survivor, Health facility)
	7	“Two weeks after completing my medication I was experiencing persistent cough every night. I was releasing sputum combined with blood every time I cough (…) I was thinking that I still had the cough–maybe I was not completely healed. That’s why I decided to go back to get a prescription.(…) I was terrified because the way I was coughing made me feel that I have Tuberculosis” (TB-survivor, Health facility)
	8	“I feel anxious because when I tell the health care providers about my situation most of them feel that I am defaulting, yet it’s not like that” (TB-survivor, Community)
	9	Interviewer: “After being healed, were you able to comply with the advice you were told back then?” TB-survivor: “The only thing I did not follow was on [moving to] a different job. You should understand from this perspective that we do go to work so that we should survive (…) I was only worried that I was exposing myself to dusty conditions which is a risk since I was working at the tobacco processing factory (…) I am planning to go to the hospital so that I can present my complaint because they say that Tuberculosis (TB) can resume sometimes (…) Somehow, I feel like this might have been caused due to my job, as I was working with Limbe Leaf.” (TB-survivor, Community)
	10	“They said that they have detected some fluids which is a sign of TB. But I was delighted to hear about that (…) I have been able to figure out the causative root of my illness which could be TB or any other disease. I was very happy at that moment” (TB-survivor, Health facility)
Counselling across the care pathway	11	“We can say that most of what is said is through experience, but to say that we have been trained in these things, we will be lying. What we learnt is about how we should counsel a person who has TB so that he or she should adhere to treatment and should not be a defaulter or that the person should not be a failure. The thing is that we learn a lot of things as we go along.” (TB-Officer, Focus group discussion) “During our briefing or training sessions that we have had there was nothing about management of recurrent TB patients that was taught.” (TB-Officer, Focus group discussion)
	12	“Soon after he had stopped taking his drugs he was not feeling well and this can be due to his reckless behaviour whereby he was smoking and drinking alcohol a lot” (TB-Officer, Focus group discussion) “They don’t adhere to their treatment and they don’t eat adequate nutritious food or they were not following what we counselled them, like not engaging in hard work. He was not following that, that is why he brought in symptoms like shortness of breath.” (TB nurse, Health facility)
	13	“When someone has finished his/her TB treatment we tell them that should you experience a similar problem, you are free to come back to the hospital, meet the doctors and tell them that I was on TB treatment, but now I’m experiencing this and that.” (TB Officer, Focus group discussion)
	14	“Their conduct in adhering to TB treatment, the nature of their job, and their adherence to ARVs, make us believe they may fall sick again.” (TB Office, Focus group discussion) “Sometimes to determine post TB patients’ health, we also look at the patient’s lifestyle. There are some who are, say, chain smokers. They may have stopped smoking during the six months of treatment, they start again and become candidates for TB retreatment. We also look at the issue of diet. (…) Where there is poor diet, where there is malnutrition, TB finds an opportunity…and because of this, we have our doubts that this one may find us again soon.” (TB Officer, Focus group discussion)
Health seeking pathways	15	“If you have one clinician against hundred patients—how are we going to see the patients; unlike one clinician against ten patients? The time you are going to spend with each patient is going to matter.” (Medical doctor, Health facility)
TB focused initial investigation	16	“When you look at the X-ray image for a TB patient you will notice that the lungs have been affected, and if he or she recovers the lung are left behind with scars. Hence this makes the interpretation difficult in someone who had once suffered from TB. The X-ray image can only be useful if there are about a number of people who are there to assess that image and come up with a conclusion. But if it is just one person assessing it, then you may end up putting someone on TB treatment, yet it is not TB.” (TB officer, Health facility)
	17	“I keep getting different judgment and treatment towards my condition. This worries me a lot…. I really feel bad, because all these three doctors had different views towards my condition, as such; this makes me feel even more worried because I don’t really know what is actually troubling me… I am not worried about getting a recurring TB, but I am only worried because I have been in this condition for a long time and I feel I am not getting any better.” (TB-survivor, Health facility referral)
	18	“We try out the patient on antibiotics (…) We will tell the patient to take it for seven days and then come later. And if the symptoms are still present, then we can re-initiate [TB] treatment.” (TB Nurse, Health facility)
Decision making around TB retreatment	19	“As the TB-Officers we trust most the GeneXpert results, because it gives an instant result. Because they are a lot of factors that are considered by the doctors [on CXR] like scars, for them to conclude if its TB or not. In our case we trust most the GeneXpert–if it shows that it has been detected then we don’t doubt about it.” (TB-Officer, Health facility) According to me, GeneXpert is very accurate because if the result for GeneXpert is positive, then I will put the patient on treatment. (Clinical officer, Health facility)
	20	“We discuss sometimes, but it all depends on the person you are working with. Some make decisions on their own; sometimes one may just decide to refer a case just to get done with it.” (Clinical officer, Health facility)
	21	“We sit down and discuss because there are some other patients who come at the facility and when they undergo the TB screening, the result tends to be negative yet they are presenting signs and symptoms for TB therefore we sit down as clinicians and nurses so as to come up with the way forward on that case so that we can unanimously agree that the patient should be put on treatment…One of the challenge that I had pointed out is about human resource. There are few health workers thus making a very good decision can be very hard.” (Clinical officer, Health facility)
	22	“The pathways don’t allow for early referral to bigger centres. The use of antibiotics is fine, I think, in a setting where you have limited resources for diagnosis, it is fine. But I would say that if you are dealing with somebody who has had pulmonary TB before and they come back with respiratory symptoms, early referral to a larger centre would be beneficial in the sense that there will be more diagnostic infrastructure (…) These complex cases, the sooner you refer them to a centre of excellence the better.”(Medical doctor, Tertiary referral hospital)
	23	“The main challenge with those that have recurring TB is that whenever we refer them to Queen Elizabeth [tertiary referral] hospital we don’t get the feedback. whether they have been diagnosed with TB or not thus we don’t do follow up.” (Clinical officer, Health facility)

TB-survivor, Health facility–TB-survivor actively health seeking and identified through TB-Officers in health facilities; TB-survivor, Community–TB-survivor not actively involved in health seeking, but identified in the community.

Healthcare seeking for recurrent symptoms was largely within the formal health system, with limited reference to traditional/spiritual treatment (Table 1, Quote 6). This biomedical approach was rooted in the belief that TB is a condition best managed within the health system, and fear that symptoms represented recurrent TB disease (Table 1, Quote 7).

Symptomatic TB survivors expressed anxiety that they would be blamed for their symptoms if they returned to care. They worried that they would be accused of poor medication adherence during the initial TB treatment episode, participation in harmful behaviours such as alcohol use or smoking, or ongoing work in harmful environments which they had been advised to avoid (Table 1, Quote 8). These fears were rooted in the counselling received during the initial TB disease episode where these exposures were framed as the cause of TB disease. In addition to fear of being blamed by others, TB-survivors expressed self-blame, with several questioning how their own behaviours might have led to recurrent illness, or expressing concern about ongoing exposure to dusts and fumes in occupations which they were unable to leave for financial reasons (Table 1, Quote 9). These exposures were framed as risk factors for TB disease only, rather than for broader respiratory disease.

Symptomatic TB-survivors returning to care experienced multiple visits for clinical review or investigations, with a lack of clear diagnosis of the cause of their symptoms. This was particularly the case for those who were sputum Xpert MTB/Rif negative on investigation. Most of our participants had received several courses of antibiotics with little perceived benefit. For several of them a diagnosis of recurrent TB disease was met with relief, with TB retreatment felt to offer renewed hope of recovery (Table 1, Quote 10).

### The post-TB care pathway

**Fig 1:**
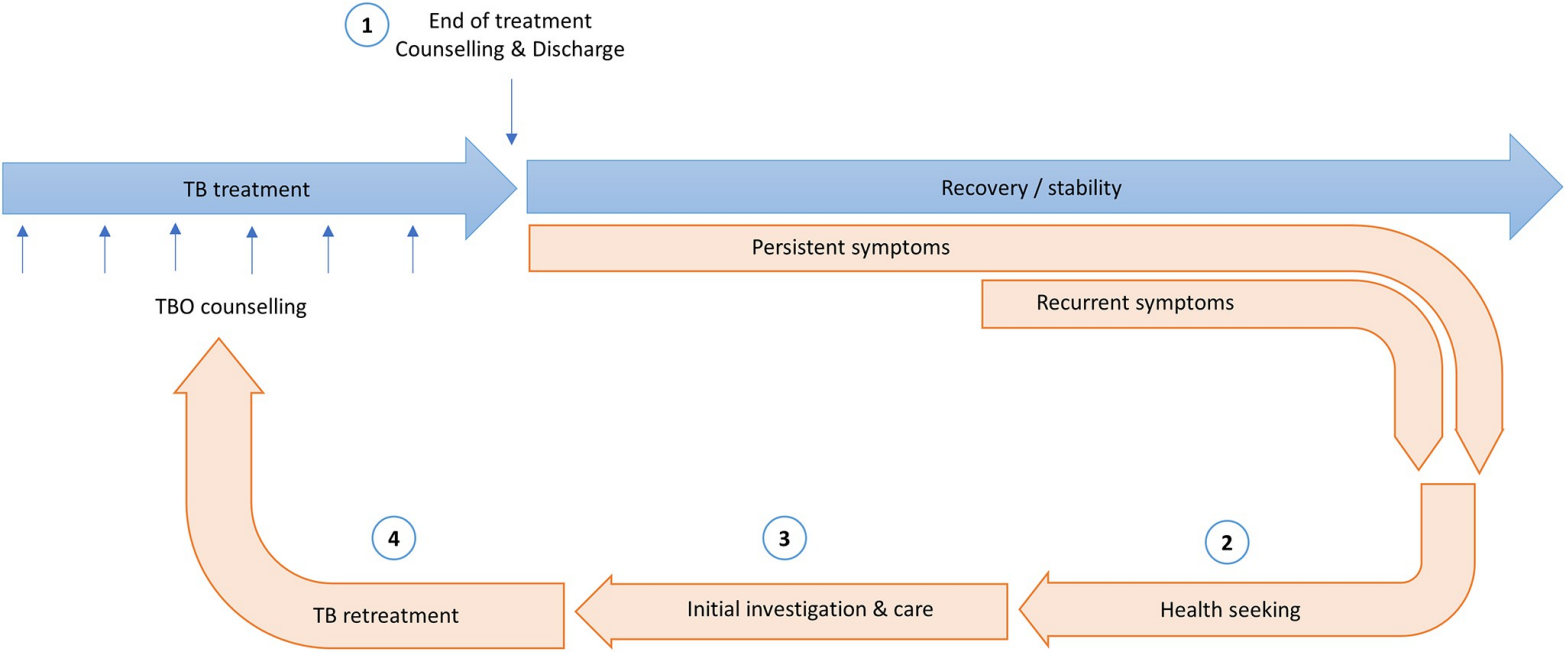
The post TB care pathway, with four critical intervention points. 1. Counselling and approaches to discharge at the end of TB treatment; 2. Health seeking pathways for return to care; 3. Initial investigation and care when re-engaging with services; 4. Decision making around TB retreatment.

We identified four time points along the post-TB pathway, which were critical in shaping patient experiences (Fig 1).

### TB counselling and discharge from care

Our data suggest limited counselling about residual symptoms at treatment completion, and a lack of follow-up for TB-survivors with ongoing symptoms or concerns.

Counselling during TB treatment in Malawi is delivered by TB-Officers, who described limited training in counselling, and on-the job experience determining the messaging used (Table 1, Quote 11). The focus of counselling was largely around a set of behaviours and exposures (e.g. poor TB treatment adherence, occupational dusts and fumes, smoking, alcohol) believed to cause TB disease, and which patients were told to avoid in order to remain well. These factors were framed as being within the control of patients, with recurrent TB disease and symptoms occurring as a result of failing to follow advice (Table 1, Quote 12). Neither the risk of re-infection after treatment completion, nor relapse occurring due to inadequate treatment were mentioned by HCWs as a potential cause of disease recurrence. Whilst TB-Officers described clearly advising patients on when and how to seek care in the event of deterioration after TB treatment completion (Table 1, Quote 13), the majority of TB-survivors did not recall receiving this advice (Table 1, Quote 5).

TB-Officers indicated that they often knew which TB-survivors were at high risk of relapse due to poor adherence during treatment, or broader social determinants of health (e.g. malnutrition, poverty, alcohol excess). However, the TB-survivors interviewed had all been discharged at treatment completion without linkage to follow-up or ongoing care to address these challenges. HCWs confirmed that these patients were usually discharged, with a sense of surrender about what would follow (Table 1, Quote 14).

### Health seeking pathways

We identified uncertainty amongst HCWs about health seeking pathways for symptomatic TB-survivors, with no agreed pathway for when and how they should return to care. The health seeking approaches described by TB-survivors were similarly heterogenous, often with initial use of over-the-counter medications in the community prior to health facility review. TB-survivors were more likely to return to TB services directly if they had established a good relationship with the TB-Officers during the initial treatment episode. However, many returned to general medical outpatient departments at health facilities, where both TB-survivors and HCWs described the limited amount of time available for assessment due to patient volume and staff shortages (Table 1, Quote 15).

### TB-focused investigations and care

Initial investigations for those who did return to care were focused on excluding recurrent TB disease and drug resistance, with little attention to broader cardio-respiratory disease.

Most HCW described initial investigation with sputum Xpert MTB/Rif and CXR, with samples sent to the reference laboratory for TB culture. TB-Officers emphasized the challenge of differentiating between PTLD and recurrent TB disease on CXR (Table 1, Quote 16), but discussed CXR findings in relation to making a TB diagnosis only. There was no suggestion that CXR was used to identify other types of cardio-respiratory disease, or of linkage of those with likely PTLD to ongoing care. HCWs noted that respiratory and cardiac diagnostics (e.g. spirometry, echocardiography) are only available to clinicians in the tertiary referral hospital. TB-survivors were occasionally referred to other medical teams for investigation of non-TB causes of cough, but described anxiety due to conflicting diagnoses and lack of communication between providers (Table 1, Quote 17).

The use of antibiotics was widespread, with a range of drugs and durations used. In the absence of clear guidelines, several clinicians described using a trial of antibiotics as part of the TB diagnostic pathway, with clinical treatment response used to inform decision making around TB retreatment (Table 1, Quote 18). Others described using antibiotics to treat bacterial infection whilst awaiting results of TB investigations.

### Varied decision making around TB retreatment

Most symptomatic TB-survivors were tested with sputum Xpert MTB/Rif, regardless of time from initial treatment completion. TB-Officers and clinicians trusted positive Xpert MTB/Rif results in order to initiate retreatment (Table 1, Quote 19). However decision making for those who were sputum Xpert MTB/Rif negative was perceived as more challenging, and the remit of clinicians and medical officers only.

Clinicians noted the difficulty of differentiating between recurrent TB disease and PTLD amongst those who are sputum Xpert MTB/Rif negative. Strategies used to manage this uncertainty included ad-hoc case discussions with colleagues, or referral to the tertiary centre for further investigation and senior clinical review, but these steps were variably used by individual clinicians (Table 1, Quote 20). Access to experienced colleagues was limited in smaller healthcare facilities (Table 1, Quote 21), and the timing of tertiary centre referral varied widely with several patients reporting to have been retreated several times before referral. There was a sense amongst senior clinicians in Blantyre that early referral for senior TB clinical review and broader investigation would improve clinical management (Table 1, Quote 22). However, HCWs noted the lack of feedback between health facilities and the tertiary centre, and concern that patients would be lost to follow up as key barriers to referral (Table 1, Quote 23). Although the potential harm of unnecessary TB retreatment was implicit in the approach described for decision making around retreatment, the risks and benefits considered were not explicitly described by HCWs, and we did not identify any specific decision-making tools in use.

## Discussion

The post-TB period is a critical part of the patient experience of TB disease. Our data highlights the negative experiences of symptomatic TB survivors, dominated by uncertainty, fear and anxiety. Our data suggest four critical practices which shape this experience including approaches to TB counselling, pathways for health seeking and return to care, approaches to early investigation and treatment, and decision making around TB retreatment. We highlight challenges at each of these points, which shape patient care and may have public health consequences such as delayed diagnosis of recurrent TB disease, missed diagnosis of broader cardio-respiratory disease, and misuse of antibiotics and TB retreatment. Our data suggest a need for guidelines to standardise and improve post-TB care (Table 2), and we identify research gaps which must be addressed to inform this (Table 3).

**Table 2:** Suggested components of guidelines to improve the care of symptomatic TB-survivors.

Time period	Guideline required
TB treatment & treatment completion	Non-stigmatising approaches to counselling about TB risk factors Nuanced approaches to counselling about occupational exposures Tools to support communication about potential long-term sequelae of TB disease Standardised guidance on the management of residual or recurrent symptoms, and when and how to return to care
Health seeking for recurrent symptoms	Standardised health seeking pathways for TB-survivors to return to care Pathways for extended follow-up of patients in whom there is concern about relapse or ongoing deterioration Community-based support (E.g. Peer support, civil society organisations) for advice and supported return to care
Investigation of symptomatic TB-survivors	Guidelines for the interpretation of Xpert MTB/Rif and Ultra in the post-TB period Guidelines for the use of CXR to identify non-TB pathology, with linkage to care Guidance for the use of antibiotics in symptomatic TB-survivors with CXR changes, and interpretation of clinical response
Decision making around TB retreatment	Decision making tools which support standardised approaches to decision making around TB retreatment Agreed criteria for referral to tertiary centre for specialist input Improved links with tertiary centres to support case discussion, referral, and feedback, in-person or digitally Linkage to non-TB cardio-respiratory services, to support broader investigation and consider alternative diagnoses

**Table 3:** Key areas for future qualitative research.

Time period	Suggested areas for exploration
TB treatment & treatment completion	Factors driving beliefs around TB risk and causality amongst patients and providers Approaches to counselling about respiratory and occupational exposures, which avoid blame and self-blame Approaches to explaining post-TB lung disease to TB patients, which maintain hope of recovery but are realistic about chronic symptoms
Health seeking for recurrent symptoms	Patient preferences towards post-TB follow up and pathways to care Experiences of stigma around residual or recurrent symptoms, and the impact of this on health seeking. Strategies supporting early return to care amongst symptomatic TB-survivors
Investigation of symptomatic TB-survivors	Opportunities and barriers to the use of cardio-respiratory investigations for symptomatic TB-survivors Factors informing antibiotic use by health care workers for symptomatic TB-survivors
Decision making around TB retreatment	Patient and provider perspectives on risk and benefits which should be considered in decision making around TB retreatment Potential role for decision making tools for TB retreatment Pathways for shared decision making and specialist referral for people with complex TB history and recurrent symptoms

Our data suggest that HCW counselling shapes how TB-survivors understand their own health and the TB disease process–the beliefs communicated by TB-survivors about the drivers of TB disease mirrored the messaging provided by TB-Officers, and several had acted (e.g. changing their diet or employment) in response to the advice received. Our data highlight several opportunities to improve the counselling provided. Firstly, current approaches emphasise the role of patient behaviours in driving treatment outcomes and relapse (e.g. TB treatment adherence, avoiding alcohol excess). This may support initial treatment success, but creates blame and guilt amongst those with recurrent symptoms. A more balanced counselling approach which encourages adherence, but also highlights the risk of chance relapse or reinfection, and discusses contextual risk factors for TB or respiratory disease (e.g. poverty, malnutrition, occupational exposures) may make it easier for patients to return to care, particularly if accompanied by broader interventions which target the drivers and manifestations of TB stigma across ecological levels [27]. Secondly, better explanations of PTLD are required–although challenging for HCW to give patients hope of a full recovery from TB disease whilst discussing long-term sequelae, failure to do this may lead to uncertainty and fear amongst patients when symptoms persist. Lastly, it is critical that patients are informed about the risk of relapse and re-infection at TB treatment completion, and given clear guidance about when and how to return to care. HCW training and access to counselling tools are needed to support this, and should be considered a core tenet of person-centred TB and respiratory care [28, 29].

Our data highlighted the lack of agreement amongst HCWs about pathways to care for symptomatic TB-survivors. Standardisation, strengthening and communication about these pathways could improve the patient experience, and support direct and early health seeking [24]. Peer support networks and input from community health workers and civil society organisations have previously been used to reduce stigma and support TB patients during treatment [30, 31], and their role in supporting TB-survivors to return to care in the post-TB period should be explored. In addition, we noted that many HCW felt that they could clearly identify TB-survivors at risk of adverse long-term outcomes following TB treatment, often due to socioeconomic factors and comorbidities. Whilst the accuracy of these predictions is not clear, it may be appropriate for health services to offer follow-up pathways for those in whom there is ongoing concern at treatment completion.

When TB-survivors do return to care, our data show that investigations focus almost exclusively on identifying recurrent TB disease and drug resistance. This is very much in keeping with national TB guidelines in Malawi and more broadly [19]. However, people with presumptive TB and symptomatic TB-survivors are at risk of non-TB cardio-respiratory disease and PTLD [32], and feasible approaches to diagnosing and managing these conditions are needed. It may be possible to use the CXRs already completed within the TB diagnostic pathway to identify non-TB diagnosis, and remote or automated radiological reading could be used to support this. Data from previous prevalence surveys in East Africa have shown a high burden of non-TB pathology on imaging suggesting a potentially high yield [33]. Future programming should inform communities about chronic respiratory disease and care in order to reduce stigma [34, 35], and improved access to respiratory diagnostics and treatment will be needed to allow the conditions identified to be addressed [36].

Lastly, our data demonstrate heterogeneous approaches to decision making around TB retreatment, with some patients receiving recurrent courses of empirical retreatment for persistent symptoms. This is a well-recognised challenge [16]. We identified several barriers to robust decision making around retreatment, including the emphasis on individual clinician led decision making, lack of time to assess returning patients, limited access to experienced colleagues for case discussions, poor communication between medical teams, and barriers to tertiary centre referral. In addition, our data showed reliance on Xpert MTB/Rif for the diagnosis of recurrent disease, even soon after TB treatment completion, despite the risk of false positive results in this time period [37]. Potential approaches to improve decision making may include decision making tools for clinicians, guidelines and pathways for tertiary centre referral and feedback, emphasis on shared decision making through case review, and the use of digital tools to support remote specialist input [38]. Given the pressure clinicians feel to provide some interventions for their patients [39], access to alternative cardio-respiratory services (e.g. chest physiotherapy) may support clinicians to avoid empirical TB retreatment or antibiotic use.

We hypothesise that a critical factor underlying the challenges outlined here is the lack of evidence-based guidelines for the management of symptomatic TB survivors. Our data suggest several possible components of such guidelines (Table 2). All would need to be supported by HCW training, with formal evaluation of clinical impact, cost, feasibility and uptake.

Our findings also highlight the lack of qualitative data around experiences and practices in the post-TB period, with a small amount of previous work focused on the TB-survivor perspective in South Africa and Zambia [18]. Our work has identified several areas where qualitative research may help to understand barriers to post-TB care from both the TB-survivor and HCW perspective (Table 3).

Strengths of this study include its focus on a neglected period of the TB cascade, the inclusion of both patient and provider perspectives, and the identification of gaps in research, training and guidelines. A range of HCW and symptomatic TB survivors were included, which allowed for rich data and triangulation. The diverse research team, many of whom have lived or worked in Blantyre, Malawi for several years, provided a range of perspectives during data analysis and an intimate understanding of the local study context. However, a large amount of post-TB research has been completed in Blantyre in recent years, and this may have shaped the perspectives and practice of both our participants and the research team. Our approach to recruiting HCW prioritised those who are frequently involved in TB or respiratory care, and may have neglected broader HCW in facilities or the community, who may be the primary contact of TB survivors. All of the TBS recruited to this study had been treated for drug sensitive disease, making our findings most relevant to this population. It will be important to compare our findings to those from research naïve sites, different HCWs, diverse patient groups, and different research teams going forward.

In summary, our data highlight the negative experiences of TB survivors with residual or recurrent respiratory symptoms after TB treatment completion. We have identified four time points at which potentially low-cost interventions could be used to improve patient care. These include more robust counselling during TB treatment and at completion, clarity around health seeking pathways for a return to care, consideration of non-TB cardio-respiratory conditions during early investigation and care, and more robust and supported decision making around TB retreatment. Our findings make a strong case for investing in research and guideline development for the post-TB period, in order to improve the long-term health and wellbeing of TB-survivors.

## Supporting information

S1 Table Research team characteristics. (DOCX)

S2 Table Factors affecting research rigour. (DOCX)

S1 Checklist (DOCX)
