# Supplementary material for: Respiratory symptoms after TB treatment completion: A qualitative study of patient and provider experiences in urban Blantyre, Malawi
Source: PLOS Glob Public Health. 2024 Sep 27;4(9):e0003436. doi: 10.1371/journal.pgph.0003436 (PMC11433068; doi:10.1371/journal.pgph.0003436)
Supplement: S1 Table — (DOCX) [file pgph.0003436.s001.docx]

S1 Table: Research team characteristics

|  | Nationality / Location during study | Occupation | Gender | Experience and training |
| --- | --- | --- | --- | --- |
| Jamilah Meghji | British / UK | Senior clinical lecturer & Respiratory physician | F | Respiratory physician and clinical academic based in the UK, with several years of research into post-TB lung disease in Blantyre, Malawi. |
| Wezi Msukwa Panje | Malawian / Malawi | Research assistant | F | Research assistant with previous training and qualitative experience on TB-focused research studies in urban Blantyre, Malawi. |
| Elizabeth Mkutumula | Malawian / Malawi | Project manager | F | Programme manager with formal training in public health and strong experience of community engagement, with experience of previous respiratory research in Blantyre, Malawi |
| Wala Kamchedzera | Malawian / Malawi | PhD student | F | PhD student with previous TB-focused research experience in Blantyre, Malawi, including use of both qualitative and quantitative methods. |
| Ndaziona PK Banda | Malawian / Malawi | Respiratory physician | M | Respiratory physician based at QECH – the 3^ry^ referral hospital for the southern region of Malawi – with extensive local clinical experience in the diagnosis and management of TB and post-TB lung disease. |
| Peter Macpherson | British / Malawi | Professor of Global Public Health | M | Epidemiologist and academic public health physician, with expertise in the epidemiology and control of infectious disease including TB /HIV, with several years living and working in Blantyre, Malawi. |
| Nora Engel | German / Netherlands | Associate Professor of Global Health | F | Senior social scientist with extensive experience of TB focused qualitative research and fieldwork in Asia and Africa. |

Interviews and focus group discussions were conducted by WMP and EM
