# Supplementary material for: Respiratory symptoms after TB treatment completion: A qualitative study of patient and provider experiences in urban Blantyre, Malawi
Source: PLOS Glob Public Health. 2024 Sep 27;4(9):e0003436. doi: 10.1371/journal.pgph.0003436 (PMC11433068; doi:10.1371/journal.pgph.0003436)
Supplement: S2 Table — (DOCX) [file pgph.0003436.s002.docx]

S2 Table: Factors affecting research rigour

| Criteria | Considerations |
| --- | --- |
| Credibility | - The study was rooted in long standing relationships between the research team and local communities and health care workers in urban Blantyre, focused around research to understand and improve TB care. The research team therefore had a good understanding of the clinical, health system, and social context of the work, when designing and developing the study aims and methods. - Pilot interviews were held to improve the format and content of the questions asked, as the start of the study - Data collection was continued to saturation - Full transcriptions and translations were obtained for all interviews and focus group discussions, with verification by the research assistant who had collected the data, who is fluent in the local language, Chichewa (WMP) |
| Dependability | - A combination of inductive and deductive approaches were used for coding, with codes developed and refined by several members of the research team (WMP, JM, EM, NE) - Comparison of findings between different forms of HCW data (IDIs and FGDs) and between different groups of TB survivors (those who were, and were not, actively engaged in health seeking) allowed for triangulation, and was used to determine areas of agreement / contrast - Discussion of data findings with TB community advisory group, TB officers, and TB survivors who had participated in the study was used for respondent validation |
| Confirmability | - Data were collected over a 1-year period, to allow time for reflection and review by the study team - The research team included a mixture of individuals with clinical/research experience in post-TB lung disease in Blantyre (JM, NPKB), those with broader TB research experience in Blantyre (WPM, EM, PM), and those with TB research experience from other settings (NE), thus bringing a range of perspectives to data analysis - Field notes and memos were kept by the research team (WMP and EM) to capture observations, and to reflect on own position and bias - Regular discussions within the research team were used to review data, discuss codes, and highlight areas of bias or uncertainty over the course of the research period (WMP, EM, JM, NE) |
| Transferability | - The significant amount of research into post-TB lung disease in Blantyre, Malawi, over the past 5-10 years may have shaped the perspectives of participants – particularly health care workers – thus limiting transferability of findings to research-naïve sites. |
